# Gender roles and intimate partner violence among female university students in Spain: A cross-sectional study

**DOI:** 10.1371/journal.pone.0259839

**Published:** 2021-11-11

**Authors:** Andrea Llano-Suárez, Alberto Lana, Ángel Gasch-Gallén, Ana Fernández-Feito

**Affiliations:** 1 Central University Hospital of Asturias, Principality of Asturias Health Service, Oviedo, Principado de Asturias, Spain; 2 Department of Medicine, School of Medicine and Health Sciences, University of Oviedo, Oviedo, Principado de Asturias, Spain; 3 Healthcare Research Area, Health Research Institute of Asturias (ISPA), Oviedo, Principado de Asturias, Spain; 4 Department of Physiatry and Nursing, University of Zaragoza, Zaragoza, Spain; University of Michigan, UNITED STATES

## Abstract

**Background:**

Traditional gender roles (GRs) have a considerable influence on relationships among couples. These can lead to negative health effects in women; however, their impact on intimate partner violence (IPV) has been less explored, especially among younger women.

**Objective:**

To explore the association between traditional GRs and several indicators of IPV on a sample of Spanish female university students involved in heterosexual dating relationships.

**Methods:**

A cross-sectional study involving female university students (n = 1,005) pursuing ten degrees (four Health Science degrees and six Social Sciences degrees). Data were collected using two validated scales: 1) the Questionnaire on the Gender Determinants of Contraception (COGANT), used to examine four traditional GRs (submissive, blind, and passive attitudes of female students, and male dominance), and 2) the Dating Violence Questionnaire-R (DVQ-R) scale, used to measure five types of IPV-behaviors (coercion, detachment, humiliation, sexual violence, and physical violence), perceived fear, entrapment, and abuse. Logistic and linear regressions were conducted to study the association between GR and a series of IPV indicators in dating relationships.

**Results:**

Traditional GRs were highly prevalent (57.0% submissive, 52.0% blind attitude, 75.7% passive, and 31.7% identified their boyfriend as being dominant). Up to 66.3% experienced some type of violent behavior. All GRs were significantly associated with IPV indicators. A submissive attitude in female students was the GR that was most strongly associated to total IPV-behavior (adjusted odd ratio [OR] = 3.18; 95% confidence interval [CI]: 2.29–4.42), followed by male dominance (OR = 2.79: 95% CI:1.71: 4.54). Both GRs were also highly associated with perceived fear, entrapment, and abuse.

**Conclusions:**

A high presence of traditional GRs was found in the relationships held by female university students, which was significantly associated with IPV indicators. Universities must adopt policies for gender equality and raise awareness on dating violence.

## Introduction

### Patriarchy and gender roles

Gender has been traditionally defined as being a social and cultural construct which determines aspects, such as the values, attitudes, and expectations that are considered appropriate for men and women [1–3]. Currently, gender refers to the self-concept developed for each person during personal growth; thereby, gender is considered a continuum construct that can change over time [4]. Gender roles (GRs) are instilled from childhood via the differential socialization and education of boys and girls, which takes place within the family environment, among friends and peers, and is conveyed by cultural conceptions in the arts, media, and religion [5–7].

Patriarchy is a system of social, economic, and political structures based on male dominance [8]. The configuration of GRs in a patriarchal society usually implies a series of demands to adapt to the expected role. In this way, pressure is exerted to maintain a system where men remain in power in the most valued spheres of life, whereas women are typically relegated to inferior or less visible positions. [1,9]. Obviously, GRs have an outstanding impact on the way in which intimate partner relationships are constructed [10]. According to traditional GRs, girls and young women are expected to configurate their attitudes and individual positions within the couple to respond to the demands of patriarchal society [3,10].

### Association between gender roles and intimate partner violence

Traditional GRs are associated with social- and health-related problems, including undesired pregnancies and sexually transmitted infections [3,10,11]. However, it is important to improve the current knowledge regarding the impact of GRs on intimate partner violence (IPV). According to previous scientific literature, social expectations about women can reinforce the silencing of some female IPV victims, their unwillingness to share experiences of violence and their difficulty in recognizing indicators of IPV. [12]. This hampers the social recognition of abuse, favoring the approval of the aggressor’s behavior and making it more likely for the victim to remain in a violent relationship [6,13]. For example, women with low self-esteem, who are insecure or submissive are more likely to stay in a violent relationship compared to empowered women [14].

However, the association between traditional GRs and IPV may be different in dating relationships among young people, as IPV during early adulthood has singular characteristics compared to relationships during adulthood.

Firstly, although the prevalence of dating violence is very high -even two to three times higher than during adulthood [15,16]- young people often have difficulty recognizing themselves as being victims of abuse [15,17–19]. For example, some types of psychological violence, such as humiliation or coercion, usually goes unnoticed by victims unless their signs are very evident [20–22]. For young adults, it is also common to believe in certain myths of romantic love, assuming, for example, that it is normal to suffer for love [17], or that controlling behaviors and jealousy are part of falling in love [20,23–25].

Secondly, another differential characteristic of IPV in youth dating relationships is that IPV tends to be bidirectional, as a person can be both the aggressor and the victim, which also hinders the recognition of abuse [2,16,17].

Thirdly, considering that young people have been exposed to traditional GRs for less time than adults, their effect on IPV may be different. Moreover, younger people might be more flexible towards the expectations they have of masculinity and femininity [26,27]. It should also be noted that the characteristics of partner relationships during youth are not usually the same as in adulthood. This means that there is a greater chance that younger people do not live together or feel financially dependent on others [15,28]. Also, the predominant types of abuse during each age group are different, as subtler, and hidden forms of violence often occur among young people [29,30].

Finally, during youth, people are forming their own identities and dating expectations about relationships and patterns of partner attachment are formed [28,31]. Thus, certain dynamics emerge within the first dating relationships, such as forms of communication, the emergence and resolution of conflicts, or exploration of sexuality, among others, influencing how people relate in adulthood [5,16,28,32]. Therefore, there is a risk that young partners who are involved in violent dating relations may see abuse as a normal part of a relationship [17,33].

### Gender roles and intimate partner violence in Spain

Over the last decade in Spain, previous research on GRs and stereotyping has identified several changes in how certain traits are viewed as typically masculine or feminine, which may be related to the dynamic character of the masculinity–femininity construct itself and the changing social roles of men and women [34]. Thus, young people have considered themselves as being more undifferentiated, both in their self-perception and in their social networks [35], as opposed to other age groups [36]. Nonetheless, it is important to closely monitor these issues, as other recent studies have highlighted clear disadvantages and social vulnerability in terms of the imposition of GRs among young women in Spain [37].

Regarding IPV, 39.7% of young women in Spain aged 16–24 years have suffered from some type of abuse throughout their lives, including psychological, physical and/or sexual violence [38]. The Spanish government has assumed the control of IPV as a matter of priority, based on its magnitude and consequences, identifying young women as a particularly vulnerable group [39]. In order to face IPV, various state measures have been developed, such as the creation of a Ministry of Equality, the declaration of a multi-state pact against male violence, the enactment of specific laws, the implementation of a national protocol for IPV prevention with coordinated actions for the health, police and judicial systems [40], and the inclusion of topics related to IPV and equality in the educational curriculum, among others. These measures have contributed towards generating an intense social rejection of IPV [41] that is reflected in the agenda of the media and in the regular celebration of social acts that condemn IPV. At the university level, programs are being developed for the primary and secondary detection of IPV [42]. Furthermore, strategies to reach gender equity and to deal with IPV have been progressively introduced throughout the educational curriculum and in numerous institutional and academic events in Spanish universities.

### Purpose of the study

Studies examining the association between GRs and IPV during dating relationships are very limited, despite of the relevance of the topic. Furthermore, an examination of this association in Spain may provide consistency to previous research involving samples with different cultural and social characteristics. In addition, focusing the research question on university students may be relevant since the educational setting should encourage reflection on IPV and it can benefit from the study results. Moreover, the group of female university students was selected based on the need to explore the relationship between GRs and IPV in a sample of women at the end of their youth and in early adulthood.

The hypothesis was that the presence of certain traditional GRs (i.e., submissive, blind and passive attitudes among female university students and the perception of domination by their male partners) increases the risk of female IPV victimization during dating relationships. Thereby, the aim of our study was to explore the association between traditional GRs and several indicators of IPV on a sample of female university of Spain in the context of heterosexual dating relationships. Moreover, we examined whether the association between traditional GRs and IPV varied across strata defined by the type of bachelor’s degree, the family history of IPV and continuity with the relationship.

## Materials and methods

### Study design and participants

A cross-sectional study was conducted on a sample of 1,005 female students enrolled in ten different Bachelor degrees of the University of *Blinded for peer review* (Spain). These Bachelor degrees included four Health Science degrees (Nursing, Physiotherapy, Medicine and Psychology) and six degrees from the department of Social Sciences (Law, Labor Relations and Human Resources, English Studies, Spanish Language and Literature, Early Childhood Educator, Primary Education). The inclusion criteria for this study consisted of the following: women over the age of 18 years, being involved in a heterosexual relationship for at least one month at some point in their life and having sexual intercourse. All participants provided written informed consent. The study was approved by the Research Ethics Committee of Principality of Asturias (Spain) (N° 140/177) and the Executive Board of the University of *Blinded for peer review* (Spain).

The University of *Blinded for peer review* is a public institution and the only university in the province of *Blinded for peer review*. Approximately half of the population in this province lives in an urban environment (i.e., cities with more than 50,000 habitants and with a density of at least 1.500 residents per km^2^). Moreover, given that there is universal access to education, healthcare and unemployment and retirement pensions, there are no major social inequalities regarding access to health, education, employment, etc. The non-Spanish ancestry population is less than 4% and the Catholic religion is the predominant religious orientation (69%). Lastly, students with different cultural and religious identities (e.g., Roma people, Evangelical, Muslim, Protestant) are a minority at this university, since the general student body is largely homogeneous. The University of * Blinded for peer review* has developed a protocol for the prevention and procedure of action in cases of moral, sexual and/or by sex, disability, sexual orientation, gender identity, beliefs, or any other reason for harassment.

### Data collection and study variables

To estimate the sample size required to conduct the study, the following parameters were set prior to recruitment: two-sided alpha of 0.05, statistical power of 0.90% and 10% percentage points of difference on IPV prevalence between groups defined by GRs. According to the results of a pilot study conducted on students from Nursing and Physiotherapy degrees (N = 203), the most frequent IPV indicator was detachment (54.7%), followed by coercion (39.9%), humiliation (25.1%), sexual (23.6%) and physical violence (8.4%). Under these assumptions, we estimated that at least 1.008 participants would need to be surveyed.

A convenience sampling method was used to recruit participants. Prior to recruitment and data collection, researchers met, reviewed, and rehearsed the procedure using standardized methods. First, participation was offered to all degree programs at the University of *Blinded for peer review*, however, we only included those in which the dean or one of the professors expressed interest. Thereafter, each professor dedicated a few minutes to the researchers during one of their lectures. Therefore, students could be studying in any of the four years of the Bachelor’s degree (six years, in the case of Medicine).

Recruitment of participants took place in the classrooms during the first trimester of 2018. The classroom was accessed either at the beginning or at the end of theoretical classes, and the subject of these classes was unrelated to the topic of study. The professors were not present during the administration of the questionnaire. After accessing the center, the study objectives and procedures were explained, inviting students to complete an anonymous self-administered questionnaire. Male students were offered the possibility of leaving the classroom or staying inside without talking to their female classmates while they completed the questionnaire. No data was collected from the students to facilitate their identification. Research staff explained to students that their participation was voluntary and confidential and solely consisted of responding honestly to the questionnaire. They were also informed that this study was independent of their academic activity and that non-participation would not have any negative effect on their academic grade. The students did not receive any financial compensation for their participation in the study. An e-mail address was included in the questionnaire to ask questions, send comments, or for students to inform of their intention to abandon the study.

Students were asked to reflect on their current dating relationship in the case that they have only been involved in one relationship, or in the most conflictive/shocking relationship if they had been involved in several relationships. The questionnaire gathered data on the respondents’ age, university studies, age of their first sexual intercourse, total number of dating relations, the continuity of the relationship with the selected partner, and IPV background in the family context. In addition, the questionnaire included two scales, the Questionnaire on the Gender Determinants of Contraception (COGANT scale) to evaluate the GR [10], and the Dating Violence Questionnaire-R (DVQ-R) to evaluate IPV [43].

The COGANT scale consists of 36 items grouped into four dimensions: relational dimension, female gender role, maternity, and care. This scale was developed by Yago Simón and Tomás Aznar in 2013 and was validated only for young Spanish women (Cronbach’s Alpha = 0.86) [10,11]. The validation of the scale involved 200 young girls and women (14–24 years old) who attended a health promotion center. The items were formulated based on verbatim quotations from the young women on the dimensions related to sexual-reproductive behavior, mainly on heterosexual relationships, romantic relationships, care, and motherhood. The scores ranged from 36 to 108 points. In our sample, the Cronbach’s Alpha was 0.83.

In this study, only the relational dimension of the COGANT scale was used (18 items), which describes a woman’s attitude towards her partner (submissive, blind, and passive), as well as women’s observations regarding the partner’s attitude towards herself (male dominance), which were considered traditional GRs. Female students were asked whether they had ever said or thought the featured statements in the selected relationship during their lifetime (e.g., “If I lose him I have nothing, I’m nothing”) (S1 Table). Each item has three response options: “no”, “sometimes”, and “yes”, which are scored with 1, 2 and 3 points, respectively (after reverse scoring some of the items which run in the opposite direction). To compare the GRs with different numbers of items, weighted scores were obtained for each GR (1 to 3 points), where 1 indicates absence of GRs, and corresponds to a more egalitarian and autonomous attitude of female students, and 3 corresponds to a great influence of GRs and loss of autonomy. Therefore, the highest score indicates greater submissiveness, blinding or passiveness of the woman, or perceived dominance on behalf of the man. Lastly, for some analyses, these scores were transformed into categorical variables. Following the “zero tolerance” criteria, it was considered that a female student displayed a submissive, blind, or passive attitude, or perceived her partner as being dominant when the weighted score was >1point. Thus, a female student was deemed to be influenced by GRs when they answered "sometimes" or "yes" to at least one item within each GR.

The DVQ-R scale evaluates the IPV via 20 behaviors grouped into five types of violence: coercion, detachment, humiliation, sexual violence, and physical violence. This scale was validated by Rodríguez-Díaz et al. in 2017 using a sample of 6,138 adolescents and young adults, of whom 60.4% were females, and 24.2% were university students (Cronbach’s Alpha = 0.85) [43]. In our sample, the Cronbach’s Alpha of DVQ-R scale was 0.94.

The items are based on a five-point Likert scale (1: “never” to 5: “almost always”), reflecting the frequency with which certain IPV behaviors occur. Each female student was asked for the frequency of occurrence of 20 violent behaviors (e.g.: “He criticizes, insults you, or yells at you”) in the selected relationship during their lifetime (S2 Table). The weighted scores were calculated for each type and for the total IPV score, therefore, the scoring ranged between 1 (absence of IPV) and 5 (maximum IPV). Following the “zero tolerance” criteria, it was considered that the woman suffered some kind of abuse when the weighted score was >1 point, i.e., if they acknowledged having suffered some type of violence "sometimes" or more frequently. In addition, the questionnaire gathers the self-perception of fear, entrapment, and abuse, based on dichotomous questions with yes/no responses.

### Data analysis

**Fig 1:**
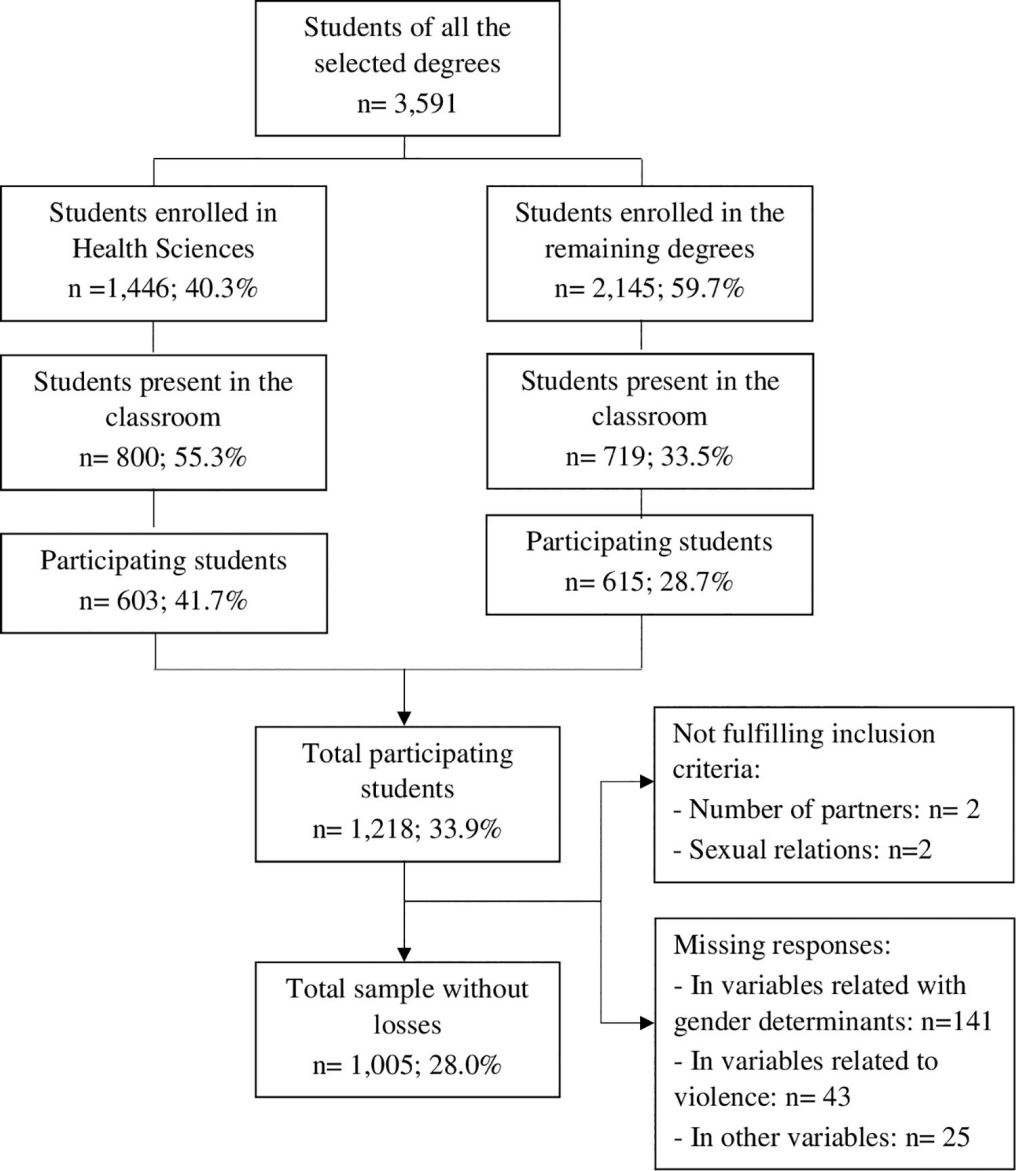
Recruitment process of the study subjects.

Of the 3,591 female university students enrolled in the selected degrees, 1,218 were recruited for the study and completed the survey. Then, we excluded 4 female students who failed to fulfill the inclusion criteria and 209 who were lacking data on certain study variables. Therefore, the final sample consisted of 1,005 participants (Fig 1). There were some differences between female university students included in the analytical sample and those who were excluded due to lacking data. Compared to included participants, those excluded were older, began sexual relations at an older age, had more dating partners and a lower frequency of continuity during problematic relationships (S3 Table).

An anonymized database was created, where a numerical code was assigned to each participant. Analyses were performed using the STATA v.15 statistical program (StataCorp, Collage Station). Only values of p<0.05 were considered statistically significant.

Qualitative variables were described using frequencies and proportions (%). Means and 95% confidence intervals (95% CI) were calculated for the quantitative variables. The unpaired t-test (dichotomous variables) or analysis of variance ANOVA (more than two groups) was used to compare the mean GR scores, based on the participants’ characteristics.

For the main analyses, we used binary logistic regressions and reported odds ratios (OR) and their 95% CI. The explanatory variables were each GR, and outcome variables were the IPV indicators, based on a dichotomous model, and the self-reported perception of fear, entrapment, and abuse. These regressions were performed according to two models. The first model was adjusted for several potential confounders, which were selected after an extensive literature review and considering the social context of the study. Thereby, the model was adjusted by age (<20, 20–22, 23–25, >25 years), degree (Health Sciences, Social Sciences), age of the first sexual intercourse (<14, 15–17, 18–20, >20 years), number of partners (1, 2, 3, >3), continuity of the relationship with the selected partner (yes, no), and family history of IPV (yes, no). Subsequently, given that there were strong correlations between the scores of each GR (S4 Table), the second model was additionally adjusted for the remaining GRs (e.g., we adjusted by “blind attitude”, “passive attitude” and “male dominance” in the analyses that referred to “submissive” attitude).

As ancillary analyses, to verify the robustness of the results, the association between the weighted scores of the GRs and the weighted scores of the DVQ-R was also examined via linear regressions. Thus, we obtained regression coefficients and their 95% CI which expressed changes in the score for each dimension of the IPV, associated with the increase of 1-point in the GRs score.

Finally, given that the ability to recognize abuse can be highly relevant for the purpose of this study, we performed several additional analyses, stratified according to the variables which indirectly reflected the students’ capacity to identify abusive behaviors. Specifically, the linear regressions were repeated and stratified according to the type of degree, the family background of IPV, and the continuity of the relationship. We considered that the following subgroups of students theoretically had a greater ability to recognize IPV: health sciences students, due to their academic training in facing violence; students who have witnessed IPV in their family and those who have decided to end the dysfunctional relationship.

## Results

### Characteristics of the female university students regarding gender roles and intimate partner violence

According to the “zero tolerance” criteria used in this study, 57.0% of students were submissive in the selected relationship, 52.0% had a blind attitude, 75.7% were passive and 31.7% perceived that their partner was dominant at some point during the selected relationship. Correlations between GR scores are shown in S4 Table. According to these results, all GRs were directly related to each other (p<0.001), notably, having a dominant partner was related with passive and submissive attitude of female students.

Regarding IPV indicators, 39.5% of the female students suffered coercion, whereas 56.0% reported detachment, 25.5% suffered humiliation, 21.3% experienced sexual violence, and 8.2% suffered physical violence at some point during the selected relationship. Overall, 66.3% suffered some type of IVP behavior. In addition, 9.3% acknowledged experiencing feelings of fear during the relationship, 24.2% felt entrapped, and 10% reported feeling abused.

Overall, the GRs included in this study (i.e., submissiveness, blinding, passivity, and male dominance) were associated with an earlier age of beginning sexual relations, a greater number of dating partners, the continuity of the problematic relationship, and having a family history of IPV (Table 1).

**Table 1:** Sampling distribution and mean scores of GRs according to the characteristics of participants (n = 1,005).

	Participants	Submissive attitude	Blind attitude	Passive attitude	Male dominance
Age	n (%)	Mean (95% CI)	Mean (95% CI)	Mean (95% CI)	Mean (95% CI)
<20 years	385 (38.3)	1.26 (1.23; 1.29)	1.44 (1.39; 1.48)	1.39 (1.36; 1.43)	1.13 (1.10; 1.16)
20–22 years	430 (42.8)	1.25 (1.21; 1.28)	1.34 (1.30; 1.37)	1.41 (1.37; 1.45)	1.14 (1.12; 1.17)
23–25 years	118 (11.7)	1.20 (1.15; 1.25)	1.22 (1.16; 1.29)	1.43 (1.36; 1.50)	1.09 (1.05; 1.13)
>25 years	72 (7.16)	1.33 (1.23; 1.42)	1.39 (1.27; 1.50)	1.52 (1.40; 1.63)	1.19 (1.11; 1.28)
p-value^a^		0.068	<0.001	0.112	0.078
**Degree**					
Health sciences	505 (50.3)	1.26 (1.23; 1.28)	1.35 (1.31; 1.38)	1.41 (1.38; 1.44)	1.13 (1.11; 1.16)
Social sciences	500 (49.8)	1.25 (1.22; 1.28)	1.39 (1.35; 1.42)	1.42 (1.38; 1.45)	1.14 (1.11; 1.16)
p-value^b^		0.689	0.148	0.794	0.710
**Age of the first sexual intercourse**					
<15 years	70 (6.97)	1.34 (1.24; 1.43)	1.49 (1.35; 1.62)	1.48 (1.37; 1.59)	1.23 (1.14; 1.33)
15–17 years	684 (68.1)	1.27 (1.24; 1.29)	1.38 (1.35; 1.41)	1.42 (1.39; 1.45)	1.14 (1.12; 1.16)
18–20 years	236 (23.5)	1.20 (1.16; 1.23)	1.30 (1.25; 1.35)	1.37 (1.32; 1.42)	1.10 (1.07; 1.13)
>20 years	15 (1.49)	1.13 (1.01; 1.25)	1.20 (1.05; 1.35)	1.44 (1.20; 1.68)	1.08 (0.99; 1.17)
p-value^a^		0.002	0.003	0.166	0.006
**Number of partners**					
1	323 (32.1)	1.20 (1.17; 1.23)	1.27 (1.23; 1.31)	1.34 (1.31; 1.38)	1.08 (1.06; 1.09)
2	330 (32.8)	1.24 (1.20; 1.27)	1.38 (1.33; 1.43)	1.41 (1.37; 1.45)	1.13 (1.10; 1.16)
3	224 (22.3)	1.30 (1.25; 1.35)	1.39 (1.33; 1.44)	1.46 (1.40; 1.52)	1.19 (0.14; 1.24)
>3	128 (12.7)	1.33 (1.26; 1.40)	1.53 (1.44; 1.62)	1.52 (1.44; 1.60)	1.21 (1.14; 1.27)
p-value^a^		<0.001	<0.001	<0.001	<0.001
**Continuity of the relationship**					
Yes	516 (51.3)	1.17 (1.15; 1.19)	1.32 (1.28; 1.35)	1.28 (1.25; 1.31)	1.06 (1.04; 1.07)
No	489 (48.7)	1.34 (1.30; 1.37)	1.42 (1.37; 1.46)	1.55 (1.52; 1.59)	1.22 (1.19; 1.25)
p-value^b^		<0.001	<0.001	<0.001	<0.001
**Family history of IPV**					
Yes	119 (11.8)	1.41 (1.33; 1.49)	1.36 (1.27; 1.45)	1.55 (1.46; 1.63)	1.26 (1.19; 1.33)
No	886 (88.2)	1.23 (1.21; 1.25)	1.37 (1.34; 1.39)	1.39 (1.37; 1.42)	1.12 (1.10; 1.14)
p-value^b^		<0.001	0.861	0.001	<0.001

GR: Gender role, CI: Confidence intervals; IPV: Intimate partner violence.

^a^p-values obtained from analysis of variance (ANOVA).

^b^p-values obtained from unpaired t-test.

### Association between gender roles and intimate partner violence

A significant association between GRs and IPV was detected when the risk of suffering IPV was studied according to dichotomous GRs variables (Table 2). The greatest contribution of GRs was for physical and sexual violence, as being submissive and perceiving the male partner as being dominant was associated with an increase up to fourfold in the risk of physical violence, whereas having a passive attitude was associated with a nearly threefold increase in sexual violence. Overall, according to fully-adjusted models, being submissive and perceiving the male partner as being dominant were the GRs which were most strongly associated with the risk of suffering IPV (OR: 3.18; 95%CI: 2.29–4.42 and OR: 2.79; 95%CI: 1.71; 4.54, respectively).

**Table 2:** Adjusted odds ratios (95% CI) for the association between GRs and IPV indicators (n = 1,005).

	Coercion	Detachment	Humiliation	Sexual	Physical	Total
**Submissive attitude**						
Model 1^a^	5.01 (3.67; 6.83) ***	4.22 (3.17; 5.63)***	4.41 (3.04; 6.38)***	5.25 (3.44; 8.02)***	8.54 (3.60; 20.2)***	5.54 (4.09; 7.51)***
Model 2^b^	2.85 (2.04; 3.99)***	2.58 (1.89; 3.53)***	2.41 (1.61; 3.59)***	2.48 (1.56; 3.96)***	3.95 (1.59; 9.82)***	3.18 (2.29; 4.42)***
**Blind attitude**						
Model 1^a^	2.22 (1.67; 2.94)***	1.79 (1.36; 2.37)**	1.89 (1.38; 2.59)**	1.66 (1.19; 2.32)	1.72 (1.02; 2.90)*	2.20 (1.65; 2.93)***
Model 2^b^	1.39 (1.01; 1.91)*	1.12 (0.82; 1.54)	1.06 (0.74; 1.52)	0.77 (0.52; 1.62)	0.82 (0.45; 1.51)	1.35 (0.97; 1.88)
**Passive attitude**						
Model 1^a^	3.81 (2.59; 5.59)***	3.47 (2.49; 4.84)***	5.02 (2.95; 8.55)***	6.15 (3.25; 11.7)***	5.13 (1.81; 14.5)***	4.50 (3.25; 6.24)***
Model 2^b^	1.96 (1.30; 2.95)**	1.96 (1.37; 2.80)**	2.51 (1.44; 4.38)**	2.76 (1.42; 5.37)**	2.18 (0.74; 6.44)	2.38 (1.67; 3.37)***
**Male dominance**						
Model 1^a^	6.61 (4.83; 9.05)***	4.03 (2.88; 5.64)***	4.35 (3.15; 6.02)***	5.95 (4.19; 8.44)***	8.71 (4.76; 16.0)***	7.50 (4.83; 11.7)***
Model 2^b^	3.18 (2.24; 4.53)***	1.59 (1.08; 2.35)*	1.88 (1.28; 2.76)**	2.48 (1.63; 3.76)***	3.94 (1.98; 7.83)***	2.79 (1.71; 4.54)***

CI: Confidence intervals; GR: Gender role; IPV: Intimate partner violence.

^a^Model 1 adjusted for age, degree, age of the first sexual relationship, number of partners, continuity with the relationship and family history of IPV.

^b^Model 2 additionally adjusted for the complementary GR.

*p-value<0.05

**p-value<0.01

***p-value<0.001.

Analogously, male dominance was the GR which most contributed to a feeling of fear, of being trapped or which led to the acknowledgment of suffering abuse in the relationship (Table 3). Regarding the attitudes of the female students in this study, submissiveness and passivity, in this order, were the GRs which most contributed to fear, entrapment and abuse. Blind attitude was not associated with IPV behaviors, nor with fear, entrapment or abuse in the fully-adjusted models.

**Table 3:** Adjusted odds ratios (95% CI) for the association between GRs and the perception of fear, entrapment and abuse (n = 1,005).

	Fear	Entrapment	Abuse
**Submissive attitude**			
Model 1^a^	8.96 (4.01; 20.0)***	4.93 (3.33; 7.32)***	8.28 (3.86; 17.8)***
Model 2^b^	3.89 (1.66; 9.12)***	2.60 (1.69; 4.01)***	3.57 (1.59; 8.01)***
**Blind attitude**			
Model 1^a^	1.54 (0.95; 2.51)	1.72 (1.24; 2.38)*	1.65 (1.02; 2.67)*
Model 2^b^	0.63 (0.35; 1.15)	0.86 (0.59; 1.27)	0.71 (0.40; 1.28)
**Passive attitude**			
Model 1^a^	3.33 (1.47; 7.53)**	5.31 (3.02; 9.32)***	5.02 (1.95; 12.9)***
Model 2^b^	1.12 (0.46; 2.69)	2.55 (1.41; 4.60)**	1.81 (0.67; 4.87)
**Male dominance**			
Model 1^a^	8.43 (4.79; 14.8)***	7.42 (5.25; 10.5)***	8.55 (4.92; 14.8)***
Model 2^b^	3.39 (1.77; 6.48)***	3.53 (2.36; 5.29)***	3.71 (1.98; 6.84)***

CI: Confidence intervals; GR: Gender role.

^a^Model 1 adjusted for age, degree, age of the first sexual relationship, number of partners, continuity with the relationship and family history of intimate partner violence.

^b^Model 2 additionally adjusted for the complementary GR.

*p-value<0.05

**p-value<0.01

***p-value<0.00.

Similar findings were observed when exploring the associations between GRs and IPV using adjusted linear regressions. Thus, we found that a 1-point increment in the scoring of the GRs was invariably associated with increases in the score of any of the types of IPV. The dominance of the male partner was the GR which most clearly contributed to the total score of the IPV, given that, for each increase of 1-point in this GR, the IPV also increased 1-point on the total score of the DVQ-R scale (regression coefficient = 1.0; 95%CI: 0.9–1.1 and 0.64; 95%CI: 0.56–0.72, when adjusting for the remaining GRs) (Table 4).

**Table 4:** Changes in the scoring of the IPV dimensions for every 1-point increase on the GR scores: Adjusted linear regression coefficients (95% CI) (n = 1,005).

	Coercion	Detachment	Humiliation	Sexual	Physical	Total
**Submissive attitude**						
Model 1^a^	0.11 (0.10; 0.13)***	0.10 (0.08; 0.12)***	0.11 (0.09; 0.12)***	0.11 (0.09; 0.12)***	0.06 (0.05; 0.07)***	0.76 (0.70; 0.83)***
Model 2^b^	0.05 (0.03; 0.08)***	0.06 (0.04; 0.08)***	0.06 (0.04; 0.08)***	0.04 (0.02; 0.06)***	0.03 (0.01; 0.04)**	0.35 (0.28; 0.42)***
**Blind attitude**						
Model 1^a^	0.05 (0.03; 0.06)***	0.03 (0.02; 0.04)***	0.11 (0.09; 0.12)***	0.03 (0.02; 0.04)***	0.01 (0.01; 0.02)*	0.24 (0.18; 0.29)***
Model 2^b^	0.01 (-0.01; 0.03)	-0.01 (-0.01; 0.01)	-0.01 (-0.01; 0.01)	-0.01 (-0.02; 0.01)	-0.01 (-0.01; 0.01)	-0.02 (-0.06; 0.02)
**Passive attitude**						
Model 1^a^	0.10 (0.08; 0.11)***	0.09 (0.07; 0.10)***	0.08 (0.07; 0.10)***	0.09 (0.08; 0.11)***	0.05 (0.04; 0.05)***	0.57 (0.51; 0.62)***
Model 2^b^	0.05 (0.03; 0.07)***	0.06 (0.04; 0.08)***	0.04 (0.02; 0.05)***	0.05 (0.03; 0.06)***	0.01 (0.01; 0.02)*	0.16 (0.10; 0.21)***
**Male dominance**						
Model 1^a^	0.12 (0.10; 0.14)***	0.08 (0.06; 0.10)***	0.12 (0.10; 0.13)***	0.14 (0.12; 0.15)***	0.08 (0.07; 0.09)***	1.00 (0.93; 1.07)***
Model 2^b^	0.04 (0.01; 0.07)**	-0.01 (-0.03; 0.02)	0.04 (0.02; 0.07)***	0.08 (0.06; 0.10)***	0.05 (0.04; 0.07)***	0.64 (0.56; 0.72)***

IPV: Intimate partner violence; GR: Gender role; CI: Confidence intervals.

^a^Model 1 adjusted for age, degree, age of the first sexual relationship, number of partners, continuity with the relationship and family history of IPV.

^b^Model 2 additionally adjusted for the complementary GR.

*p-value<0.05

**p-value<0.01

***p-value<0.001.

Finally, in the additional analyses, the associations were maintained across all strata, although they were of greatest magnitude among female students who theoretically had a greater capacity to identify IPV. Specifically, female students of Health Sciences, with a family history of IPV and who decided to end the problematic relationship (S5 Table).

## Discussion

The aim of this study was to explore the association between GRs and IPV among a sample of Spanish female university students. According to the results of our cross-sectional study, GRs were directly associated with IPV indicators. Specifically, being submissive and perceiving their partner as being dominant were the GRs with the greatest explanatory power.

In our study, we observed a high percentage of female students with passive, submissive and blind attitudes, which seems to confirm the fact that, even today, traditional GRs and patriarchy still prevail [3,6,9,16,20,34]. Furthermore, one out of every three female students perceive their male partner as being domineering, suggesting that the historically established male role is perpetuated [5,16,44]. Other studies have also found that traditional GRs still exist in Spain, although they have considerably changed in the last decades. Moya and Moya-Garófano [45] have recently compared gender stereotypes in Spain in two different time periods (1985 and 2018). According to their analysis, people perceived an increase of men with female-linked GRs and vice-versa. These changes could be more evident in Spain than in other European countries, due to the unequal starting point in Spain [46].

Despite the efforts made in recent years by the political powers and by Spanish society to achieve gender egalitarianism, it is still necessary to develop initiatives to eliminate traditional GR and to mitigate their deleterious effects, especially among young couples. It is a priority to advocate for gender equality policies at all levels. The educational system, from childhood to university, must take an active role in the socialization and transmission of values that lead to the banishment of GR. However, the responsibility should not fall solely on the educational system; society, impelled by political powers, should strive to achieve equal relations between men and women. Comprehensive and cross-cutting action plans are needed to achieve this goal within a global framework of action with concrete recommendations, since, in many cases, isolated programs are implemented that have a limited effect (e.g., workshops on affective sexual education with a gender perspective). Regarding victimization, the percentage of female students who suffered some type of IPV was lower than other previous reports [15,18,21,47]. These findings may indicate a decline in the prevalence of IPV in recent years in Spain among young people, perhaps due to a positive effect of government measures to end IPV. In addition, there is currently greater awareness, sensitivity and/or visibility of this problem. Although IPV has always been considered an intimate problem affecting couples, it is increasingly seen as a social problem, which favors an attitude of rejection towards people who practice IPV. Detachment was the type of violence most frequently suffered by students. This was related to psychological violence, representing the most normalized and prevalent violence, which is also difficult to identify, as it is intangible and often occurs in a subtle manner [20,32,33,48]. Therefore, it should be emphasized that certain detached behaviors are also part of IPV, while reflecting unequal dating relationships. It is necessary to empower female students to be able to identify silent indicators of IPV early on, such as detachment.

In addition, an important percentage of female students in this study reported having suffered physical or sexual violence in their relationships. Physical violence appears to be more easily recognizable, as the percentage of female students who have suffered physical violence and those who identified themselves as having been abused was similar. This seemingly demonstrates that teenagers relate physical violence with abuse. However, sexual violence is often overlooked, as it is not identified as abuse, but rather as “going with the flow” or “indulging the intimate partner,” something which is normalized in relationships between young people. Many women do not feel like they are victims of sexual abuse, even when they have maintained an undesired sexual relationship, as they find it difficult to recognize the abuse [49].

The female students in our study reported feelings of fear to a lesser extent than other studies [50]. This may be because they associated fear with physical violence [18], as the percentages of both findings were similar. This is possibly because physical violence is the most obvious and the one that most endangers women’s safety. The other types of violence, as they are more discreet, go more unnoticed or are not acknowledged to the same extent. Furthermore, this study features young couples who typically do not have children, are not economically dependent, do not live together and are highly educated. This can reduce the conflicts, and thus, their experience of fear is lessened.

Following the aim of our study, we have observed that both the submissive attitude of female students and the perception of male dominance bear the greatest relation with suffering IPV. This seems to be based on the ideas of patriarchy and machoism which generate dominant men who typically subjugate women [51]. The dominance and control of men over women is something which is accepted and has been socially normalized for many decades [1,5,9,44]. In our study, the perception of male partners as being dominant generated the most violent and visible forms of abuse, and hence, the greatest alarm. However, subtler forms of domination are often invisible. It is important to note that female submissiveness and male dominance are usually interrelated, as dominant men tend to relate to submissive women, and vice versa, due to their traditional beliefs regarding GR. This means that female students can easily feel underestimated in these relations [35,52]. A recent study on 441 students form a Turkish Vocational School of Health Services found a statistically significant negative association between the score indicating equalitarian attitudes and the score for attitudes towards IPV (Pearson’s correlation coefficient = -0.67; p-value: 0,04) [53]. Therefore, students supporting traditional GRs could be more permissive with psychological and physical violence during dating relationships, which is in line with our findings. Additionally, a study on 759 Chilean university students also found that traditional GRs, defined by high stereotypical and low transcendent attitudes, predicted victimization by coercion, detachment, humiliation and physical violence among female students [54]. Although authors recognized the low explanatory power of GRs, their results are congruent with our findings, derived from analyses adjusted for several confounders.

The association between the weighted 1-point increment on the GR scale and the frequency of IPV is noteworthy, as this finding represents a change of approximately 20% (this scale ranges from 1 to 5 points). From the point of view of health consequences, our results have major clinical/social relevance for the lives of female students as this represents a moderate increase in the frequency of the violent behaviors that they suffer.

### Implications for practice and/or policy

The kind of relationships that shape a submissive woman and a dominant male are usually explained by the impositions placed on the structure of gender dynamics, which favor and legitimize this standpoint, arising from a reproduction of traditional GRs [55]. Therefore, on a global scale, it is of upmost importance to encourage gender equality interventions from infancy in order to decrease the current differences [1,5]. To act on this matter, the gap between research and policy needs to be reduced. It is imperative to disseminate the results of research on GRs and IPV among stakeholders, especially educational, social, and health institutions.

Concerning the implications for university training, by implementing training based on gender equality, inequalities could be reduced. Such training could be conducted in group interventions addressing issues such as the analysis of the GRs present in relationships during youth, young female student’s empowerment, or training in the early detection of risk indicators for violence. Through these actions, students will not only adopt skills to detect violence, but they will also acquire skills to enhance communication and conflict resolution. This may lead to building healthier partner relationships and decreasing the contribution of GRs to the violent behaviors suffered by female students. Ultimately, reducing tolerance to violence, and most likely improving female student’s health at all levels (physical, sexual, and psychological), considering that several studies have shown that IPV negatively impacts a victim’s health [3,5,9,16,32].

Although our previous recommendations have been focused on female students, because they are our study population, it is important to remember that IPV is a problem involving the society at large, in which men are also responsible, and with whom it is necessary to take action [56]. Obviously, if interventions and policies were focused only on female students, rather than on the society at large, this would deepen sexism and such interventions would be doomed to failure. An in-depth exploration of GRs in socialization and their relationship with IPV in men seems to be a necessary starting point for designing interventions to enable more egalitarian relationships and eradicate IPV. In any case, it is well known that tolerance of IPV and traditional GRs work synergistically to increase the risk for the perpetration of dating violence among males [57,58]. As a society, we cannot turn our back on this problem, which implies an analysis of the roles and couple dynamics of young people which may perpetuate relationships based on gender inequality [59].

In general, this study invites a reflection on the implications of IPV against female students. From a broad perspective, a change is necessary in the information conveyed by the media, literature, music, and other agents as, currently, they continue to perpetuate traditional gender stereotypes and roles [5,6,60]. In the university environment, this reflects the need to include a mainstream gender perspective [61], raising awareness on IPV by implementing dedicated action plans. Concretely, university lecturers can be key drivers to achieve gender egalitarianism by incorporating this perspective into their teaching activity and into the curricular content as a transversal competence [62]. Moreover, universities should strongly contribute towards supporting healthy dating relationships, through the promotion of gender equality and female empowerment. In addition, greater collaboration with other pre-university educational institutions would be desirable, since GRs linked to dating relationships are first notable during adolescence. Both educational levels could collaborate in new lines of research related to GRs and IPV. Furthermore, it would be necessary to promote qualitative research to better understand how young people perceive the influence of GRs and their possible involvement in issues related to health and/or interpersonal relationships, not only between couples, but also between peers or at the family level.

### Strengths and weaknesses

A main strength was the fairly large sample, including female students from several university degrees. Another strength was the selection of relevant confounders, such as the number of partners, continuity of the relationship and the history of IPV. Moreover, we performed various ancillary analyses, using different approaches, to verify the robustness of the results.

However, the study also had several limitations. First, the cross-sectional nature of the data collection hampers the establishment of directionality in the association between the variables under study. Thus, it would be desirable to design and conduct longitudinal studies from adolescence to adulthood to detect changes in the GRs and/or the prevalence of IPV with the participation of men and women of different sociodemographic characteristics, educational levels, and/or sexual orientations.

Second, only female students with heterosexual relationships were included in this study as the questionnaire on GRs is directed at these types of relationships. Therefore, we were unable to gather information on female students who were involved in other types of relationships. Moreover, given that the study was conducted on a sample of university students, findings cannot be extrapolated to other collectives. Despite this, we believe that these findings may help estimate what occurs among similar young samples of students at other universities. Nevertheless, we are aware of the importance of extending this research to young people of both sexes, with different sexual orientations, in a non-university environment (both pre-university students and those who opt for professional training and do not go to university), or other ethnic minorities.

Third, the fact that the female students were asked to base their answers on the most conflictive partner or the one that had marked them the most is a further limitation because, in the case of students who have had several partners, this choice is subjective. In addition, if this relationship occurred some time ago, the answers may be affected by a memory bias. The fact that no information was obtained regarding when this conflictive relationship took place does not allow us to establish differences between those that took place during adolescence and those that occurred during youth.

Fourth, overall, participation in this study was low, considering that the percentage of students who participated represented approximately 35% of the students enrolled in the degrees under study. This was mainly due to the low levels of attendance in some degrees (e.g., law degree). Moreover, there were some differences between female university students included in the study and those excluded due to lack of data. However, we believe that the large sample and the inclusion of students from several degrees and different fields of science allows us to establish a reliable association.

Fifth, we did not consider whether students had already attended classes or seminars about IPV in the degrees in which IPV is part of the curriculum, such as Medicine, Nursing, Psychology or Law. This is relevant because knowledge about IPV can modify the perception of the study variables.

Sixth, as the questionnaires were completed in the classroom, some female students may not have responded with complete honesty due to the presence of their male peers. Nonetheless, they were asked to remain silent, to answer truthfully and not to share information or comments with other students and/or friends. Moreover, in most cases, the questionnaire was administered at the end of the class, thereby male peers were almost never present in the classroom while the female students completed the form. In addition, the study could also be limited by a response bias, due to the possible tendency to respond inaccurately, especially in relation to indicators of sexual abuse. To reduce response bias, research staff insisted on the importance of obtaining honest answers and on the guarantee of anonymity.

Future studies addressing this topic may further the reduce response bias using online questionnaires to be completed at home, although this procedure could also reduce the response rate. Lastly, this study had a volunteer bias, meaning that the female students who did not wish to participate may be different to those who did. However, we believe that it is more likely that the volunteer nature of this study leads to an underestimation of the prevalence and association of these GR, rather than an overestimation.

## Conclusions

The presence of GRs in the dating relationships of female university students from Spain was very high. Moreover, GRs were clearly associated with IPV indicators. The most determinant GRs were submissiveness on behalf of the women and perceiving the male partner as being dominant.

## Supporting information

S1 Table GRs of the relational dimension included in the COGANT scale. (DOCX)

S2 Table IPV-behaviors included in the DVQ-R scale grouped in five types of violence. (DOCX)

S3 Table Differences between female university students included and excluded from the analytic sample due to the questionnaire lacking data (n = 1,218). (DOCX)

S4 Table Correlation coefficients (p-values) between GRs scores. (DOCX)

S5 Table Changes in scoring of the IPV dimensions for every 1-point increase on the GR scores (linear regression coefficients, adjusted model) according to degree, family history of IPV and continuity with the selected relationship. (DOCX)

S1 Data (XLSX)
