# Supplementary material for: Gender roles and intimate partner violence among female university students in Spain: A cross-sectional study
Source: PLoS One. 2021 Nov 11;16(11):e0259839. doi: 10.1371/journal.pone.0259839 (PMC8584681; doi:10.1371/journal.pone.0259839)
Supplement: S1 Table — (DOCX) [file pone.0259839.s001.docx]

**S1**. GRs of the relational dimension included in the COGANT scale

| **Submissive attitude** |
| --- |
| I think I’m pregnant but I haven’t told him because I don’t want to worry him |
| If I lose him I have nothing, I’m nothing |
| I felt sorry for him and I had sex with him although I didn’t want to |
| When he asks for forgiveness, I feel so sorry for him that I go back to him |
| When my boyfriend doesn’t have much sexual desire, I suspect that he’s interested in another girl |
| **Blind attitude** |
| With this one it will be different, we have been together for two months but we are a stable couple |
| When I’m in love I don’t think about taking care for myself during sexual intercourse |
| I knew that he had cheated on other girls but I was sure that it would be different with me |
| **Passive attitude** |
| I feel as supported by him as he is by me, I think we both put the same amount into this relationship |
| Since we have been together I have stopped going out with my friends |
| I think I’m very important to him |
| I always tell myself I won’t do it, but, in the end, I do it again |
| When I drink a lot, I behave very differently sexually |
| **Male dominance** |
| When I have sex with him, I feel he doesn’t take that much care of me |
| If I insist on the use of condoms, he goes crazy, screams, loses it, and I stop insisting because otherwise it’s worse |
| When he’s your boyfriend he gets jealous and he doesn’t let you do anything anymore |
| I fear that if I tell him I don’t like what he wants us to do, he will leave me |
| He doesn’t put on the condom and I don’t speak because I don’t want to argue |

GR: gender role
