# Supplementary material for: Gender roles and intimate partner violence among female university students in Spain: A cross-sectional study
Source: PLoS One. 2021 Nov 11;16(11):e0259839. doi: 10.1371/journal.pone.0259839 (PMC8584681; doi:10.1371/journal.pone.0259839)
Supplement: S2 Table — (DOCX) [file pone.0259839.s002.docx]

**S2.** IPV-behaviors included in the DVQ-R scale grouped in five types of violence.

| **Coercion** | |
| --- | --- |
|  | ‘‘Tests’’ your love, setting traps to ﬁnd out if you are cheating |
|  | Talks to you about relationships he/she imagines you have |
|  | Has physically kept you from leaving |
|  | Invades your space (listening to a loud music when you are studying, listening your phone calls. . .) |
| **Detachment** | |
|  | Is a good student, but is always late at meetings, does not fulﬁl his/her promises, and is irresponsible |
|  | Does not acknowledge any responsibility regarding the relationship or what happens to both of you |
|  | Has ignored your feelings |
|  | Stops talking to you or disappears for several days, without any explanation, to show their annoyance |
| **Humiliation** | |
|  | Criticizes you, underestimates the way you are, or humiliates your self-esteem |
|  | Ridicules your way of expressing yourself |
|  | Has ridiculed or insulted your beliefs, religion or social class |
|  | Ridicules or insults you for the ideas you uphold |
| **Sexual abuse** | |
|  | You feel compelled to have sex as long as you don’t have to explain why |
|  | Insists on touching you in ways and places which you don’t like and don’t want |
|  | You feel forced to perform certain sexual acts |
|  | Forces you to undress even if you don’t want to |
| **Physical abuse** | |
|  | Has beaten you |
|  | Has slapped your face, pushed or shaken you |
|  | Has thrown blunt instruments at you |
|  | Has hurt you with an object |

IPV: Intimate Partner Violence; DVQ-R: Dating Violence Questionnaire-R
