# Supplementary material for: Gender roles and intimate partner violence among female university students in Spain: A cross-sectional study
Source: PLoS One. 2021 Nov 11;16(11):e0259839. doi: 10.1371/journal.pone.0259839 (PMC8584681; doi:10.1371/journal.pone.0259839)
Supplement: S3 Table — (DOCX) [file pone.0259839.s003.docx]

**S3.** Differences between female university students included and excluded from the analytic sample due to the questionnaire lacking data (n=1,218).

|  |  | **Included** | **Excluded** |
| --- | --- | --- | --- |
|  | | **n (%)** | **n (%)** |
| **Participants** | | 1,005 (82.5) | 213 (17.5) |
| **Age** | |  |  |
| <20 years | | 385 (38.3) | 78 (36.6) |
| 20-22 years | | 430 (42.8) | 82 (38.5) |
| 23-25 years | | 118 (11.7) | 22 (10.3) |
| >25 years | | 72 (7.2) | 31 (14.6) |
| p-value^a^ | |  | 0.006 |
| **Degree** | |  |  |
| Health sciences | | 350 (34.8) | 78 (36.6) |
| Social sciences | | 655 (65.2) | 135 (63.4) |
| p-value^a^ | |  | 0.618 |
| **Age of the first sexual intercourse** | |  |  |
| <15 years | | 70 (7.0) | 10 (4.7) |
| 15-17 years | | 684 (68.0) | 116 (54.5) |
| 18-20 years | | 236 (23.5) | 62 (29.1) |
| >20 years | | 15 (1.5) | 25 (11.7) |
| p-value^a^ | |  | <0.001 |
| **Number of partners** | |  |  |
| 1 | | 323 (32.1) | 61 (28.6) |
| 2 | | 330 (32.8) | 64 (30.1) |
| 3 | | 224 (22.3) | 32 (15.0) |
| >3 | | 128 (12.7) | 56 (26.3) |
| p-value^a^ | |  | <0.001 |
| **Continuity of the relationship** | |  |  |
| Yes | | 516 (51.3) | 82 (38.5) |
| No | | 489 (48.7) | 131 (61.5) |
| p-value^a^ | |  | 0.007 |
| **Family history of IPV** | |  |  |
| Yes | | 119 (11.8) | 27 (13.4) |
| No | | 886 (88.2) | 175 (86.6) |
| p-value^a^ | |  | 0.544 |

IPV: intimate partner violence

^a^p-values obtained from the chi-square test
