# Supplementary material for: Gender roles and intimate partner violence among female university students in Spain: A cross-sectional study
Source: PLoS One. 2021 Nov 11;16(11):e0259839. doi: 10.1371/journal.pone.0259839 (PMC8584681; doi:10.1371/journal.pone.0259839)
Supplement: S4 Table — (DOCX) [file pone.0259839.s004.docx]

**S4.** Correlation coefficients (p-values) between GR scores.

|  | **Submissive attitude** | **Blind attitude** | **Passive attitude** | **Male dominance** |
| --- | --- | --- | --- | --- |
| **Submissive attitude** | - |  |  |  |
| **Blind attitude** | 0.29 (<0.001) | - |  |  |
| **Passive attitude** | 0.51 (<0.001) | 0.33 (<0.001) | - |  |
| **Male dominance** | 0.75 (<0.001) | 0.54 (<0.001) | 0.84 (<0.001) | - |
| **Mean (SD)** | 1.25 (0.33) | 1.37 (0.44) | 1.41 (0.39) | 1.14 (0.28) |

GR: gender role
