# Supplementary material for: Gender roles and intimate partner violence among female university students in Spain: A cross-sectional study
Source: PLoS One. 2021 Nov 11;16(11):e0259839. doi: 10.1371/journal.pone.0259839 (PMC8584681; doi:10.1371/journal.pone.0259839)
Supplement: S5 Table — (DOCX) [file pone.0259839.s005.docx]

**S5.** Changes in scoring of the IPV dimensions for every 1-point increase on the GR scores (linear regression coefficients, Adjusted model) according to degree, family history of IPV and continuity with the selected relationship.

|  | | **Coercion** | **Detachment** | **Humiliation** | **Sexual** | **Physical** | **Total** |
| --- | --- | --- | --- | --- | --- | --- | --- |
| **Health sciences^a^** | |  |  |  |  |  |  |
|  | Submissive attitude | 0.11 (0.09; 0.14)*** | 0.08 (0.06; 0.11)*** | 0.11 (0.09; 0.13)*** | 0.11 (0.09; 0.14)*** | 0.05 (0.04; 0.06) *** | 0.84 (0.75; 0.93)*** |
|  | Blind attitude | 0.05 (0.03; 0.07)*** | 0.03 (0.01; 0.05)* | 0.03 (0.01; 0.05)* | 0.03 (0.01; 0.04)** | 0.01 (0.01; 0.02)* | 0.28 (0.20; 0.36)*** |
|  | Passive attitude | 0.11 (0.09; 0.13)*** | 0.09 (0.07; 0.11)*** | 0.08 (0.06; 0.10)*** | 0.09 (0.08; 0.11)*** | 0.03 (0.02; 0.05)** | 0.56 (0.48; 0.68)*** |
|  | Male dominance | 0.13 (0.10; 0.16)*** | 0.07 (0.04; 0.10)*** | 0.12 (0.10; 0.15)*** | 0.16 (0.13; 0.18)*** | 0.06 (0.05; 0.08)*** | 1.12 (1.02; 1.22)*** |
| **Social sciences^a^** | |  |  |  |  |  |  |
|  | Submissive attitude | 0.11 (0.09; 0.14)*** | 0.11 (0.09; 0.14)*** | 0.11 (0.09; 0.14)*** | 0.10 (0.08; 0.12)*** | 0.07 (0.06; 0.09)*** | 0.71 (0.63; 0.79)*** |
|  | Blind attitude | 0.04 (0.02; 0.06)** | 0.03 (0.01; 0.05)* | 0.04 (0.02; 0.05)** | 0.03 (0.02; 0.05)** | 0.02 (0.01; 0.03)* | 0.19 (0.12; 0.27)*** |
|  | Passive attitude | 0.09 (0.07; 0.11)*** | 0.09 (0.07; 0.11)*** | 0.08 (0.07; 0.10)*** | 0.09 (0.08; 0.11)*** | 0.05 (0.04; 0.07)*** | 0.58 (0.51; 0.65)*** |
|  | Male dominance | 0.12 (0.09; 0.15)*** | 0.09 (0.06; 0.12)*** | 0.11 (0.08; 0.13)*** | 0.12 (0.10; 0.14)*** | 0.09 (0.08; 0.11)*** | 0.90 (0.81; 0.99)*** |
| **Without family history of IPV^b^** | |  |  |  |  |  |  |
|  | Submissive attitude | 0.12 (0.10; 0.14)*** | 0.10 (0.08; 0.13)*** | 0.11 (0.10; 0.13)*** | 0.11 (0.09; 0.12)*** | 0.05 (0.04; 0.06)*** | 0.66 (0.60; 0.72)*** |
|  | Blind attitude | 0.05 (0.03; 0.06)*** | 0.03 (0.01; 0.04)* | 0.03 (0.02; 0.04)** | 0.02 (0.01; 0.04)* | 0.01 (0.01; 0.02)* | 0.19 (0.14; 0.24)*** |
|  | Passive attitude | 0.09 (0.08; 0.11)*** | 0.09 (0.07; 0.10)*** | 0.08 (0.06; 0.09)*** | 0.09 (0.07; 0.10)*** | 0.03 (0.02; 0.04)** | 0.49 (0.44; 0.54)*** |
|  | Male dominance | 0.13 (0.10; 0.15)*** | 0.09 (0.06; 0.11)*** | 0.13 (0.11; 0.15)*** | 0.09 (0.07; 0.10)*** | 0.07 (0.05; 0.08)*** | 0.89 (0.82; 0.96)*** |
| **With family history of IPV^b^** | |  |  |  |  |  |  |
|  | Submissive attitude | 0.09 (0.05; 0.13)*** | 0.08 (0.05; 0.12)*** | 0.09 (0.05; 0.12)*** | 0.10 (0.07; 0.14)*** | 0.10 (0.07; 0.13)*** | 1.10 (0.90; 1.30)*** |
|  | Blind attitude | 0.05 (0.01; 0.09)** | 0.02 (0.01; 0.06)* | 0.04 (0.01; 0.08)* | 0.05 (0.02; 0.09)** | 0.04 (0.01; 0.07)* | 0.46 (0.20; 0.73)*** |
|  | Passive attitude | 0.11 (0.07; 0.15) )*** | 0.10 (0.06; 0.13)*** | 0.10 (0.06; 0.13)*** | 0.13 (0.09; 0.16)*** | 0.10 (0.07; 0.13)*** | 0.99 (0.75; 1.22)*** |
|  | Male dominance | 0.11 (0.07; 0.16) )*** | 0.07 (0.03; 0.12)** | 0.08 (0.04; 0.12)*** | 0.12 (0.07; 0.16)*** | 0.13 (0.10; 0.16)*** | 1.34 (1.11; 1.57)*** |
| **Does not continue with the partner^c^** | |  |  |  |  |  |  |
|  | Submissive attitude | 0.11 (0.08; 0.13)*** | 0.08 (0.06; 0.10)*** | 0.12 (0.10; 0.14)*** | 0.11 (0.09; 0.13)*** | 0.06 (0.05; 0.08)*** | 0.93 (0.84; 1.03)*** |
|  | Blind attitude | 0.05 (0.03; 0.07)*** | 0.03 (0.02; 0.05)** | 0.05 (0.03; 0.06)*** | 0.04 (0.02; 0.06)** | 0.02 (0.01; 0.03)* | 0.34 (0.25; 0.44)*** |
|  | Passive attitude | 0.10 (0.08; 0.12)*** | 0.07 (0.05; 0.09)*** | 0.08 (0.07; 0.10)*** | 0.10 (0.08; 0.12)*** | 0.05 (0.04; 0.06)*** | 0.67 (0.59; 0.76)*** |
|  | Male dominance | 0.11 (0.08; 0.13)*** | 0.07 (0.05; 0.10)*** | 0.11 (0.09; 0.14)*** | 0.13 (0.11; 0.15)*** | 0.07 (0.06; 0.09)*** | 1.04 (0.94; 1.13)*** |
| **Continues with the partner^c^** | |  |  |  |  |  |  |
|  | Submissive attitude | 0.13 (0.10; 0.15)*** | 0.14 (0.11; 0.18)*** | 0.09 (0.07; 0.12)*** | 0.09 (0.07; 0.11)*** | 0.05 (0.04; 0.06)*** | 0.42 (0.36; 0.48)*** |
|  | Blind attitude | 0.04 (0.02; 0.06)** | 0.02 (0.01; 0.04)* | 0.02 (0.01; 0.03)* | 0.02 (0.01; 0.03)* | 0.01 (0.01; 0.02)* | 0.09 (0.05; 0.14)*** |
|  | Passive attitude | 0.10 (0.07; 0.12)*** | 0.13 (0.10; 0.15)*** | 0.08 (0.06; 0.10)*** | 0.08 (0.06; 0.10)*** | 0.03 (0.02; 0.04)** | 0.34 (0.29; 0.39)*** |
|  | Male dominance | 0.21 (0.16; 0.26)*** | 0.13 (0.08; 0.19)*** | 0.11 (0.07; 0.15)*** | 0.17 (0.14; 0.21)*** | 0.10 (0.07; 0.12)*** | 0.74 (0.65; 0.84)*** |

IPV: intimate partner violence. GR: gender role

^a^Model adjusted for age, age of the first sexual relationship, number of partners, continuity with the relationship and family history of IPV.

^b^Model adjusted for age, degree, age of the first sexual relationship, number of partners and continuity with the relationship.

^c^Model adjusted for age, degree, age of the first sexual relationship, number of partners and family history of IPV.

*p-value<0.05; **p-value<0.01; ***p-value<0.001
